# Supplementary material for: Effect of Operational Parameters on the Removal of Carbamazepine and Nutrients in a Submerged Ceramic Membrane Bioreactor
Source: Membranes (Basel). 2022 Apr 14;12(4):420. doi: 10.3390/membranes12040420 (PMC9030045; doi:10.3390/membranes12040420)
Supplement: Supplementary file 1 [file membranes-12-00420-s001.zip › membranes-1657924-supplementary.pdf]

membranes

MDPI

Article

Effect of Operational Parameters on the Removal of Carbamazepine and Nutrients in a Submerged Ceramic Membrane Bioreactor

hanh-Chau Dao [1,2], Chih-Chi Yang [1], Ku-Fan Chen [1] and Yung-Pin Tsai [1,*]

1 Department of Civil Engineering, National Chi Nan University, Nantou Hsien 54561, Taiwan; daokhanhchau07@gmail.com (K.C.D.); chi813@gmail.com (C.C.Y.); kfchen@ncnu.edu.tw (K.F.C.)
2 Dong Nai Technology University, Bien Hoa, Dong Nai 810000, Vietnam
* Correspondence: yptsai@ncnu.edu.tw; Tel.: +886-49-2910960 (ext. 4121)

Citation: Dao, K.C.; Yang, C.-C.; Chen, K.-F.; Tsai, Y.-P. Effect of Operational Parameters on the Removal of Carbamazepine and Nutrients in a Submerged Ceramic Membrane Bioreactor. *Membranes* **2022**, *12*, 420. https://doi.org/10.3390/membranes12040420

Academic Editors: Michael O. Daramola, Ahmad Fauzi Ismail

Received: 14 March 2022
Accepted: 7 April 2022
Published: 14 April 2022

Publisher's Note: MDPI stays neutral with regard to jurisdictional claims in published maps and institutional affiliations.

Copyright: © 2022 by the authors. Submitted for possible open access publication under the terms and conditions of the Creative Commons Attribution (CC BY) license (https://creativecommons.org/licenses/by/4.0/).

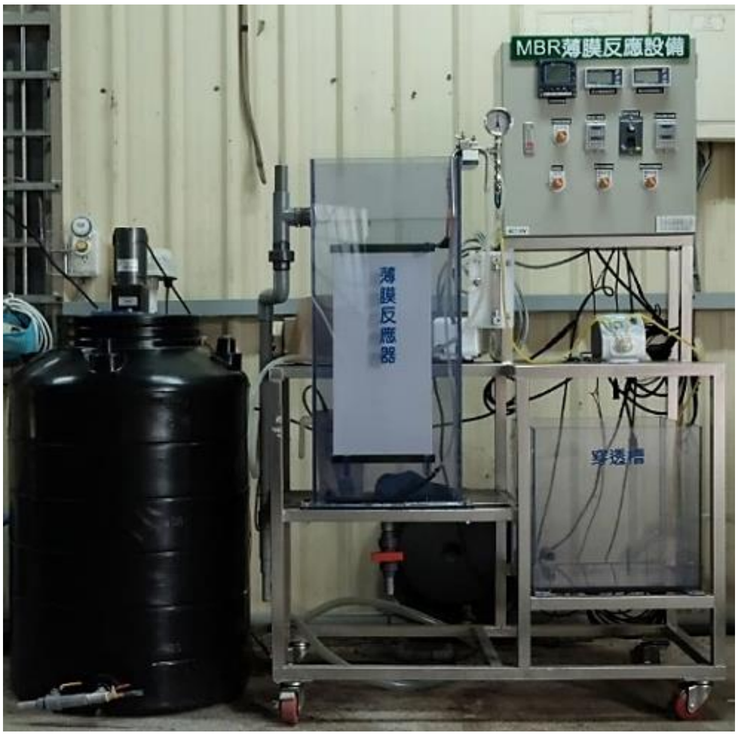

Figure S1. MBR experimental setup in practice.

Table S1. Physico-chemical and pharmacological properties of CBZ.

Chemical structure	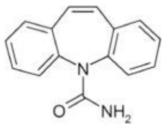
Molecular formula	$C_{15}H_{12}N_2O$
Molecular weight	236.27 g/mol
Drug Classes	Anticonvulsants
Water solubility	18 mg/L at 25 °C
Log Kow (octanol-water partitioning)	2.45
Henry's law constant	1.09×10^{-5} Pa m3 /mol (25 °C)
pKa	13.9
Melting point	189–193 °C

Biological Half-Life	35 to 40 hours after one dose of carbamazepine extended-release formulations
Elimination of half-life (environmental)	328 d
Appearance	White, light yellowish powder

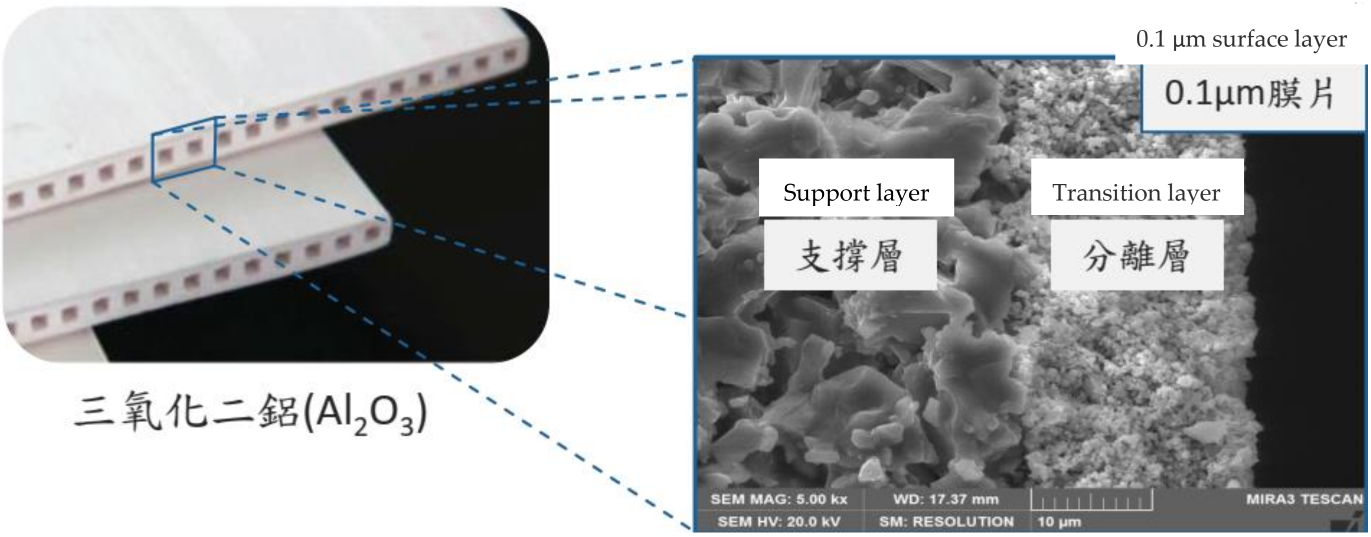

Figure S2. SEM photo of section part of flat-sheet ceramic membrane

Table S2. Experimental values of trans-membrane pressure (TMP).

		**Factor 1**	**Factor 2**	**Factor 3**	**TMP**
Std	Run	A:DO	B:HRT	C:SRT	kPa
		mg/L	h	days	
9	1	3.5	18	10	9.1
7	2	1.5	24	15	13.4
4	3	5.5	24	5	10.1
8	4	5.5	24	15	10.7
6	5	5.5	12	15	9.5
2	6	5.5	12	5	9.3
10	7	3.5	18	10	8.8
1	8	1.5	12	5	12.8
11	9	3.5	18	10	8.7
5	10	1.5	12	15	12.6
3	11	1.5	24	5	12.1

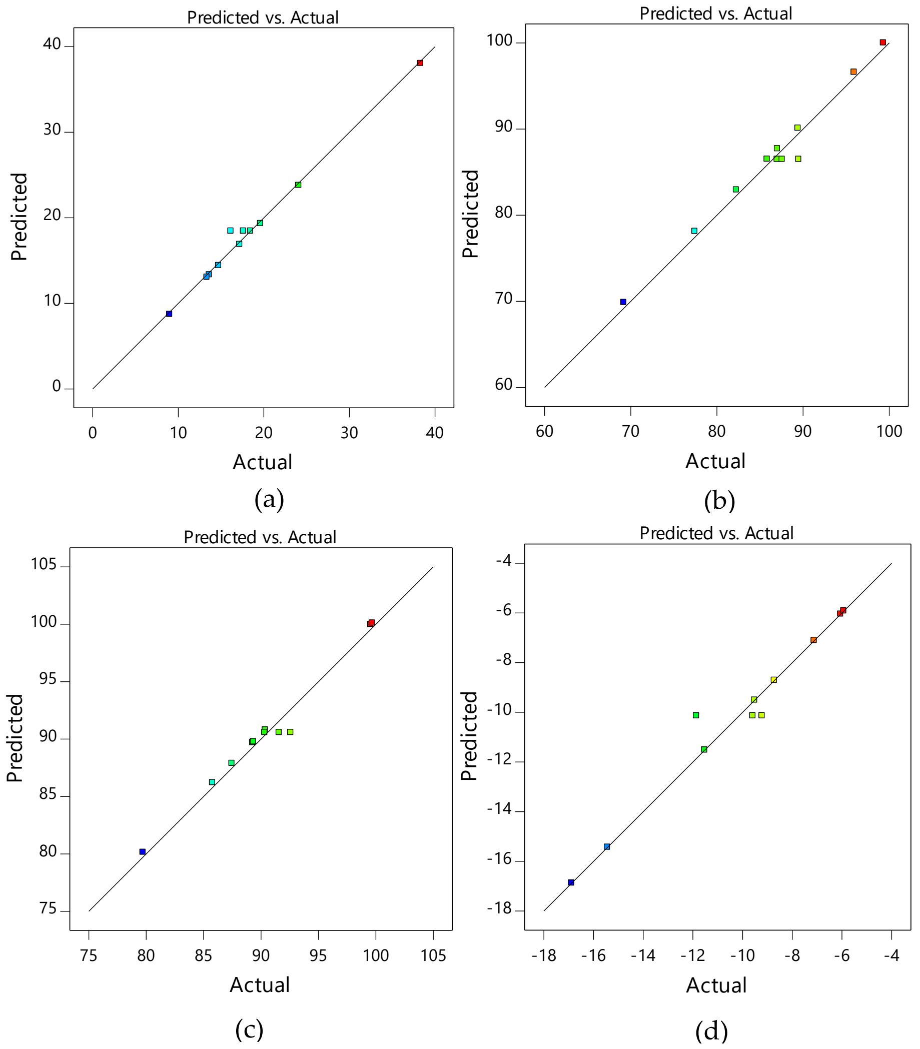

Figure S3. Comparison of predicted and actual values of: (a) CBZ, (b) COD, (c) ammonia, and (d) phosphorus removal efficiency.

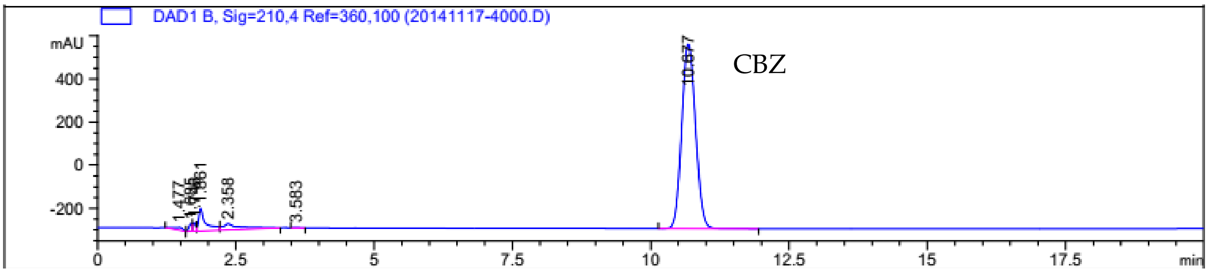

Figure S4. HPLC – DAD chromatogram of CBZ standard containing 100 μg/mL at 210 nm wavelength.

Table S3. Average influent and effluent concentrations (± SD) of CBZ, COD, ammonia, and phosphorus through experiments.

**Number**	**CBZ (ug/L)**		**COD (mg/L)**		**Ammonia (mg/L)**		**Phosphorus (mg/L)**	
	Influent (n=3)	Effluent (n=3)	Influent (n=3)	Effluent (n=3)	Influent (n=3)	Effluent (n=3)	Influent (n=3)	Effluent (n=3)
1	67.77 ± 2.43	55.77 ± 2.15	432.89 ± 30.97	55.11 ± 15.75	58.90 ± 12.55	5.14 ± 3.67	13.64 ± 0.22	14.94 ± 0.14
2	91.30 ± 7.45	73.01 ± 1.11	517.78 ± 50.92	90.67 ± 17.64	59.94 ± 2.44	5.76 ± 0.76	14.65 ± 0.09	15.92 ± 0.07
3	83.77 ± 6.95	69.10 ± 2.58	462.89 ± 19.53	2.89 ± 1.92	69.36 ± 1.37	0.29 ± 0.18	16.14 ± 0.05	18.63 ± 0.09
4	82.51 ± 5.13	70.19 ± 0.79	628.89 ± 38.49	25.11 ± 5.09	63.53 ± 0.90	0.18 ± 0.01	15.24 ± 0.22	17.81 ± 0.04
5	94.12 ± 6.99	85.55 ± 5.40	437.33 ± 8.82	61.78 ± 10.18	58.23 ± 2.40	6.13 ± 1.89	15.02 ± 0.15	16.75 ± 0.21
6	105.20 ± 2.78	90.77 ± 4.45	374.00 ± 15.28	39.56 ± 6.94	60.82 ± 1.70	8.63 ± 0.85	15.09 ± 0.08	16.52 ± 0.35
7	84.70 ± 2.71	71.00 ± 4.66	491.78 ± 9.62	60.67 ± 14.53	67.86 ± 2.78	6.50 ± 1.35	15.49 ± 0.58	17.31 ± 0.32
8	84.81 ± 5.56	64.31 ± 3.45	573.33 ± 66.67	128.44 ± 6.94	41.98 ± 13.29	7.92 ± 0.74	14.87 ± 0.27	15.77 ± 0.21
9	99.22 ± 7.58	80.79 ± 5.83	474.00 ± 20.00	49.56 ± 1.92	63.78 ± 7.99	4.64 ± 0.67	14.89 ± 0.22	16.26 ± 0.31
10	82.12 ± 17.55	70.88 ± 1.99	494.00 ± 14.53	151.78 ± 8.39	57.53 ± 0.50	7.19 ± 2.02	15.25 ± 0.14	16.15 ± 0.05
11	85.75 ± 14.09	52.69 ± 8.07	451.11 ± 38.49	58.44 ± 6.94	66.03 ± 1.92	7.05 ± 0.73	13.68 ± 0.34	14.64 ± 0.29
